# Supplementary material for: Accumulation Mechanism and Risk Assessment of Artemisia selengensis Seedling In Vitro with the Hydroponic Culture under Cadmium Pressure
Source: Int J Environ Res Public Health. 2022 Jan 21;19(3):1183. doi: 10.3390/ijerph19031183 (PMC8834386; doi:10.3390/ijerph19031183)
Supplement: Supplementary file 1 [file ijerph-19-01183-s001.zip › ijerph-1487540-supplementary.pdf]

# Accumulation mechanism and risk assessment of *Artemisia selengensis* seedling in vitro with the hydroponic culture under cadmium pressure

**Tao Tang [1,2], Wei Kang [2,3,*]Mi Shen [2], Lin Chen [1,2], Xude Zhao [2], Yongkui Wang [2], Shunwen Xu [4], Anhuai Ming [4], Tao Feng [1], Haiyan Deng [2], Shuqi Zheng [2]**

[1] School of Resources and Environmental Engineering, Wuhan University of Science and Technology, Wuhan, 430080, China; tangtaowkd@163.com (T.T.); Chenlin1735@163.com (L.C.); fengtaowhu@163.com (T.F.)
[2] Hubei Provincial Key Laboratory of Mining Area Environmental Pollution Control and Remediation, Hubei Polytechnic University, Huangshi 435003, China; 223004@hbpu.edu.cn (M.S.); zhaoxude@hbpu.edu.cn (X.Z.); 204055@hbpu.edu.cn (Y.W.); bx_ollie08051005@163.com (H.D.); Zhengshuqi2021@163.com (S.Z.)
[3] College of Environmental Engineering, Wuhan Textile University,Wuhan, 430200,China; kangwei@hbpu.edu.cn (W.K.)
[4] Huangshi Vegetable Industry Development Center,Huangshi 435003, China; xunshunwen2021@163.com (S.X.); minganhuai@163.com (A.M.)
* Correspondence: kangwei@hbpu.edu.cn; Tel.: +86-15072077233

**Supplementary Material**

**List**

1. Figure S1: The growth of *A. selengensis* tissue culture seedlings after 40 days of cultivation.
2. Table S1: Translocation factor (TF) of *A. selengensis* shoots and leaves.
3. Figure S2: Cd content (Dry weight) of *A. selengensis,* (a): root, (b): stem, (c): leaf.

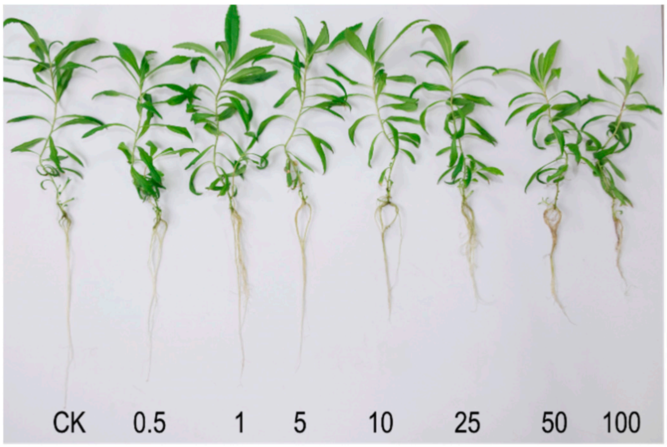


**Figure S1.** The growth of *Artemisia selengensis* tissue culture seedlings after 40 days of cultivation. From left to right, the concentration of Cd treatments are 0 μM, 0.5 μM, 1 μM, 5 μM, 10 μM, 25 μM, 50 μM and 100 μM.

**Table S1.** Translocation factor (TF) of *A. selengensis* shoots and leaves.

| Cd Concentration(μM) | Day 10 | | Day 20 | | Day 30 | | Day 40 | |
|---|---|---|---|---|---|---|---|---|
| | stem | leaf | stem | leaf | stem | leaf | stem | leaf |
| 0.5 | 0.77±0.10 | 0.18±0.02 | 0.47±0.03 | 0.21±0.01 | 0.34±0.01 | 0.17±0.01 | 0.25±0.02 | 0.15±0.02 |
| 1 | 0.72±0.06 | 0.25±0.09 | 0.50±0.04 | 0.23±0.01 | 0.32±0.03 | 0.22±0.02 | 0.27±0.01 | 0.20±0.01 |
| 5 | 1.06±0.04 | 0.32±0.01 | 0.57±0.04 | 0.20±0.01 | 0.42±0.04 | 0.23±0.04 | 0.30±0.02 | 0.23±0.01 |
| 10 | 0.99±0.03 | 0.39±0.01 | 0.60±0.02 | 0.24±0.01 | 0.40±0.09 | 0.28±0.06 | 0.40±0.03 | 0.23±0.02 |
| 25 | 1.01±0.04 | 0.37±0.01 | 0.51±0.01 | 0.20±0.04 | 0.41±0.02 | 0.18±0.01 | 0.36±0.01 | 0.19±0.02 |
| 50 | 0.55±0.02 | 0.09±0.01 | 0.36±0.03 | 0.18±0.02 | 0.32±0.06 | 0.12±0.02 | 0.29±0.01 | 0.12±0.01 |
| 100 | 0.34±0.02 | 0.08±0.01 | 0.26±0.03 | 0.14±0.02 | 0.24±0.02 | 0.16±0.02 | 0.26±0.01 | 0.15±0.01 |

The value shown is mean ± S.D.

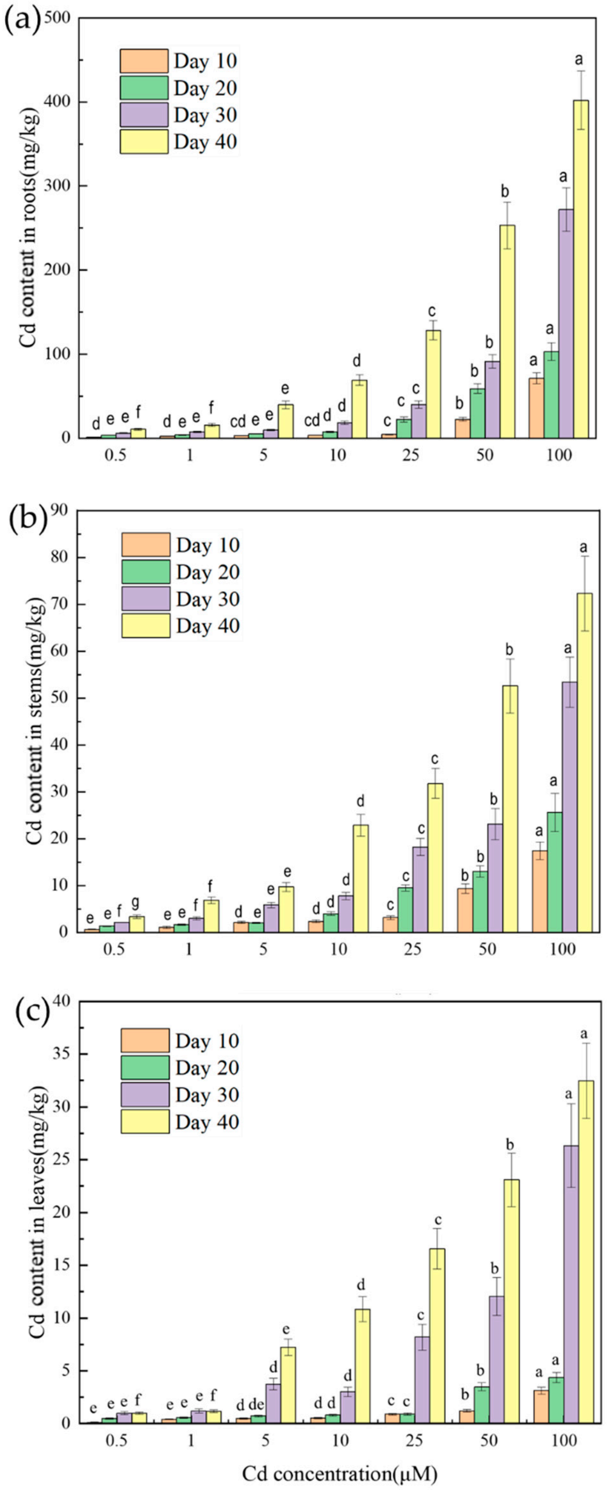


**Figure S2**. Cd content (Dry weight) of *A. selengensis,* (a): root, (b): stem, (c): leaf. The results are means ± SD (n = 3), Lower letters mean significant differences ($p < 0.05$) between different Cd concentrations at the same treatment time.
